# For a Greater University

**Published:** 1908-01

**Authors:** 


**A Monthly Review of Medicine and Surgery.**

*EDITOR:*

WILLIAM WARREN POTTER, M. D.

All communications, whether of a literary or business nature, books for review and exchanges, should be addressed to the editor 238 DELAWARE AVE., BUFFALO, N. Y.

VOL. LXIII. JANUARY, 1908. No. 6

### For A Greater University.

BUFFALO is putting up a strenuous fight for a greater university. In other words, the friends of university extension for the city of Buffalo are displaying an activity which deserves success. Vice Chancellor Charles P. Norton the leader of the university forces, is displaying the qualities of great generalship in the struggle to establish a college of liberal arts, the only department lacking to complete the cycle necessary to the establishment of a university in fact, as well as in name, for the greater city of Buffalo.

It may have seemed to some persons that the movement had come to a standstill, but it would appear from recent events that Mr. Chancellor Norton has been resting on his oars for a time preliminary to a second heat in the great race. Tuesday, December 17, 1907, was a great day for the greater university of greater Buffalo and it seems proper to name it, greater University day. In the forenoon, a hearing was had before the supervisors in relation to the proposition to exchange a portion of the present almshouse site for a property in the town of Clarence, known as the Schultz farm, the object being in securing this exchange to erect university buildings where the almshouse property is now located.

It would appear that two propositions are involved in this scheme: the one being, to convince the supervisors that it is desirable to move the almshouse into the country; and the other, to persuade the county legislature to donate the vacated site to the university authorities. The hearing was largely attended by professional and business men,—clergymen, doctors, lawyers, merchants, bankers, and citizens generally. In numbers and quality it indicated a strong movement in favor of the grant. Speakers in favor of the exchange were first heard, among whom were Alderman Callanan, Principal Fosdick of the Masten Park high school, Miss Jane Meade Welch, John F. Mueller, Alfred Paget, Alfred Hurrell, Thomas Templeton, Rev. B. S. Ferrall, Alfred Harrison, Raymond Brown, Henry W. Sprague, Wilbur E. Houpt, John W. Williams, Dr. Lucien Howe, George S. Metcalfe, Frank H. Coffran, and Louis Wright Simpson.

A few speakers appeared in opposition, but no effective arguments were presented on that side of the question. The supervisors' committee had prepared a favorable report but it was voted by the full board to postpone final action for one week, the ayes being 26, and noes 22.

In the evening of the same day the Chamber of Commerce held its annual dinner at the Ellicott Club, at which the speaking was devoted to the subject of a greater university. Again Vice Chancellor Norton was heard to good effect and his forceful speech, couched in graceful rhetoric, told of earnest devotion to the cause. Dr. A. V. V. Raymond, former president of Union College, now pastor of the first presbyterian church in this city, made an eloquent plea in favor of the establishment of a college of arts, maintaining that though there were enough country colleges, there were not an adequate number in the centers of population. Carleton Sprague, Esq., spoke eloquently in favor of a greater university and then Mr. Henry Ware Sprague offered a resolution urging the supervisors to adopt the report of its committee releasing the almshouse site for the purposes named, the resolution being adopted by unanimous vote. And so ended greater university day.

